# Traditional Therapies Used to Manage Diabetes and Related Complications in Mauritius: A Comparative Ethnoreligious Study

**DOI:** 10.1155/2016/4523828

**Published:** 2016-04-21

**Authors:** M. Fawzi Mahomoodally, A. Mootoosamy, S. Wambugu

**Affiliations:** ^1^Department of Health Sciences, Faculty of Science, University of Mauritius, 230 Réduit, Mauritius; ^2^Department of Veterinary Anatomy and Physiology, University of Nairobi, Nairobi 30197 00100, Kenya

## Abstract

Religious communities from Mauritius still rely on traditional therapies (TT) for primary healthcare. Nonetheless, there is still a dearth of scientific information on TT used by the different religious groups to manage diabetes and related complications (DRC). This study aimed to gather ethnomedicinal knowledge on TT used by the different religious groups against DRC. Diabetic patients (*n* = 95) and traditional healers (*n* = 5) were interviewed. Fifty-two plant species belonging to 33 families and 26 polyherbal formulations were documented to manage DRC. The most reported DRC was hypertension (*n* = 36). Leaves (45.2%) and juice (36%) were the most cited mode of preparation of herbal recipes. Plants which scored high relative frequency of citation were* Citrus aurantifolia *(0.55) and* Morinda citrifolia *(0.54). The cultural importance index showed that* Ocimum tenuiflorum*,* Cardiospermum halicacabum*,* Camellia sinensis,* and* Ophiopogon japonicas* were the most culturally important plants among Hindu, Muslim, Christian, and Buddhist community, respectively. Hindu and Muslim community showed the highest similarity of medicinal plants usage (Jaccard index = 95.8). Seven animal species distributed over 4 classes were recorded for the management of DRC. Plants and animals recorded as TT should be submitted to scientific studies to confirm safety and efficacy in clinical practice and to identify pharmacologically active metabolites.

## 1. Background

Diabetes mellitus, generally termed as diabetes, is a chronic metabolic disorder of the endocrine system characterised by abnormalities in carbohydrates, protein, and fat metabolism [1–3]. The incidence of diabetes mellitus continues to soar exponentially in both developed and developing countries leading to an increase in the cost of management/treatment of the disease and its related complications. Diabetes mellitus is a global epidemic currently affecting more than 371 million people and the death toll from the disease rose to 1.5 million in 2012 [4, 5]. The World Health Organisation has argued that diabetes will be the 7th leading cause of death in 2030 [6].

Diabetes mellitus is one of the most important health issues in Mauritius with a prevalence of 24.5% in 2015 [7]. The International Diabetes Federation reported that in 2015 there were 220,000 cases of diabetes in Mauritius and the number of cases of diabetes in adults that are undiagnosed was found to be 113100 [7]. Alterations in carbohydrates, protein, and fat metabolism entail an increase in blood glucose level which causes long-term devastating complications in many organs of the body [8]. Prolonged uncontrolled hyperglycemic level leads to macrovascular complications (coronary artery disease, peripheral arterial disease, and stroke) and to microvascular complications (diabetic nephropathy, neuropathy, and retinopathy) [9]. Complications related to diabetes are the major cause of disability and mortality among the Mauritian diabetic population.

Nowadays, diabetes is managed with oral hypoglycemic agents and insulin. Though the efficacy of these treatments is irrefutable, they have to be given throughout the lifetime of the patient and entail numerous potential side effects, namely, hypoglycemic coma and hepatorenal disturbances [10, 11]. Hence, there is a growing interest in traditional therapies mostly because of the less frequent side effects associated with them as compared to conventional medicines.

**Figure 1:**
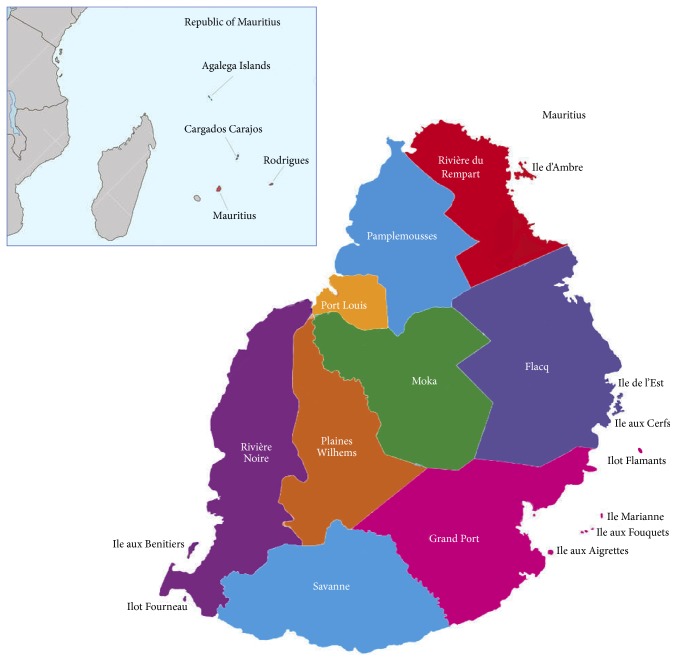
Map of Mauritius indicting the study area (spread over 9 main districts).

Mauritius is a tropical island located in the southern hemisphere in the middle of the Indian Ocean. Mauritius is bestowed with a rich variety of medicinal flora, fauna, and cultural diversity. The volcanic island of Mauritius lies in the middle of the Indian Ocean (Figure 1) with coordinates 57°30′ east and 20°20′ south. Mauritius has an area of 1,865 km^2^ and about 43% of the area is allocated to agriculture. Mauritius enjoys a mild tropical climate, characterised by a warm humid summer between November and April and a cool dry winter between June and September whereby October and May are the transition months. Mean summer temperature is 24.7°C and mean winter temperature is 20.4°C [12]. Mauritius has a rich heritage of indigenous and endemic plants. During the past, allopathic medicine was not easily available for the local population and the use of traditional medicine was therefore necessary in order to alleviate signs and symptoms of diseases. Nowadays, healthcare facilities are within the reach of everybody; nonetheless, traditional medicine continues to remain active in the lives of the local population.

The multicultural society of Mauritius encompasses descendants of Indian indentured labourers, Chinese shopkeepers, African slaves, and British and French colonisers. The Indo-Mauritians community (Hindus and Muslims) represents the majority of the population followed by the Christian community and the Sino-Mauritians community. The Hindu community is subdivided into several distinct religious and sociocultural groups. The main religious groups are the Hindi or Bhojpuri speaking people constituting 40.2% of the total population and 76.5% of all Hindus. The Tamils are the second largest ethnic community (13.9%), while Telugus (5.6%) and Marathis (4%) represent smaller minorities within the overall Hindu community. The Hindus have a common language (Bhojpuri), the same regional origin (Uttar Pradesh and Bihar), and religious practices and rituals [13]. The official language in Mauritius is the English language but “Creole” is the native language of the island and is mostly used in informal settings. Mauritius is reputed worldwide for the peaceful harmony which prevails in the island among the different great religions of the world, namely, Hinduism, Christianity, Islam, and Buddhism. In Mauritius, traditional therapies are paramount to panoply of ailments treatment/management offering profound therapeutic benefits and the indigenous communities rely heavily on them to meet their medical needs. Though allopathic medicine is the primary form of healthcare in Mauritius, some patients prefer traditional medicine for the treatment/management of a number of human diseases. The rising costs of synthetic drugs have fueled the interest of the local population in traditional medicine usage, thereby reintroducing such therapies as a novel emerging form of health aid. Mauritius is endowed with a number of tropical rainforests which are rich repositories of a diverse range of invaluable medicinal plants and animal species. Recently, Mauritius has become the arena of a number of ethnopharmacological field studies conducted by various workers which have emphasised mostly medicinal plants and animals among the Mauritian population and have led to several publications [14, 15]. Nonetheless, none of these studies have addressed the patterns of similarity and dissimilarity of medicinal plants and animals usage among the different religious communities present in the island. Heinrich et al. [16] reported that most studies on medicinal plants focus on the role of these plants within one particular ethnic group and little emphasis has been given to the comparison of medicinal plant species among various cultures. Moreover, an analysis of medicinal plants usage must be carried out in order to understand the patterns of use intra- and interculturally. However, studies on cross-cultural analysis of medicinal plants usage are lacking in Mauritius. Therefore, the current study specifically seeks to bring in the limelight of the scientific community the documentation of traditional remedies used among the diabetic patients in Mauritius. We also attempted to identify the most culturally important medicinal plants and animals in each religious group, compare the use of plant and animal species interculturally, and examine how the different religious groups present in the island value traditional remedies in their daily lives in their quest for sound health.

## 2. Methods

### 2.1. Data Collection

**Figure 2:**
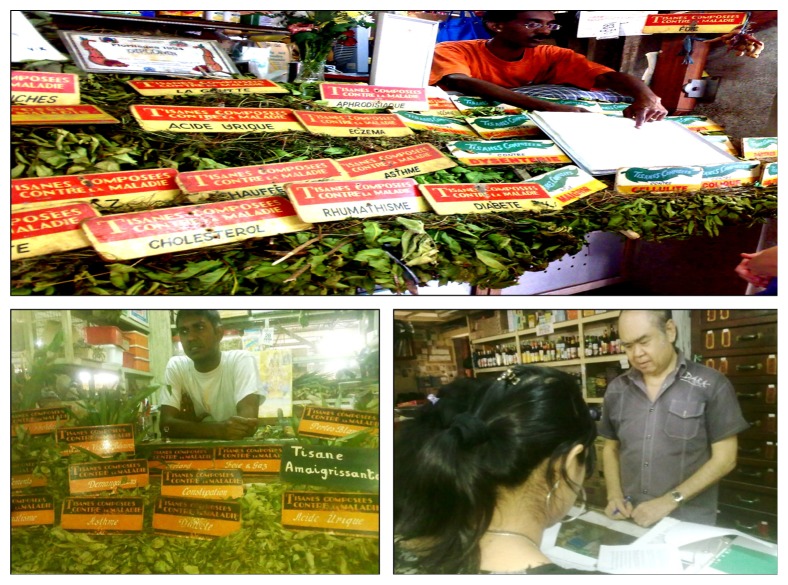
Interview with traditional healers.

The project was approved by the Faculty of Science, University of Mauritius, Mauritius. A total of 100 key informants (27 Hindus, 24 Muslims, 26 Christians, and 23 Buddhists) were interviewed from June to August, 2015. Data was collected from key informants, through face-to-face interviews, using a semistructured questionnaire (supplementary file in Supplementary Material available online at http://dx.doi.org/10.1155/2016/4523828). Traditional information was sought from diabetic patients older than 30 years based on the assumption that the mature population is better versed in traditional knowledge. Moreover, participants should be users of traditional medicine and formally diagnosed to be diabetic by their treating physician. During the course of the study, 12 field trips were carried out in different regions of the island. The interview was performed in “Creole,” the native language of the Mauritian population. The questionnaire developed for the survey consisted of both close- and open-ended questions. Participants were informed about the purpose of the survey and a prior informed consent form was dully signed by the participants before the interview was carried out. The traditional healers were interviewed using the same questionnaire. The interviews were performed in health centers, home visits, markets, and Chinese shops (Figure 2). Figure 1 illustrates the different regions where the survey was carried out.

The questionnaire comprised three main parts: Parts A, B, and C. Part A consisted of demographic data which included age, gender, level of education, occupation, income, and religious belief. Part B of the questionnaire consisted of information about the herbal remedies used to manage diabetes and related complications, the local vernacular name of the plant, the method of preparation, the dosage, the route of administration, and the duration of treatment. Part C was based on animal-based remedy used to manage diabetes and related complications, the local vernacular name of the animal, the method of preparation, the dosage, the route of administration, and the duration of treatment.

### 2.2. Collection and Identification of Medicinal Plants

During the field visits, when a remedy was mentioned by the traditional healer or diabetic patient, where possible, the participant was encouraged to show us a sample of the remedy which was collected* in situ* and photographed. The collected sample was then identified by local botanist. Our local repository database was updated whereby plant samples were assigned a collection number for future reference and data mining. Data obtained during the survey was cross-checked (local names/scientific names) according to a locally published book by Gurib-Fakim and Brendler [17]. Scientific names of plant species were identified according to the International Plant Name Index (IPNI: http://www.ipni.org/).

### 2.3. Ailments Categories

Based on the information obtained from the key informants in the study area, all the reported ailments were classified into 9 categories based on published scientific literature from Riaz [18], ADA [19], Yadav et al. [20], Ginsberg et al. [21], and Bodansky et al. [22]. The categories were diabetic angiopathy, diabetic nephropathy, eye diseases, diabetic neuropathy, infections and wounds, hypertension, skin complications, diabetic dyslipidemia, and diabetes.

### 2.4. Data Analysis and Ethnobotanical Indexes

The indigenous medicinal information of plant and animal species was analyzed using different quantitative indexes.

#### 2.4.1. Relative Frequency of Citation

Relative frequency of citation is calculated as follows: relative frequency of citation = FC/*N*, where FC is the number of informants mentioning the use of the species and *N* is the number of informants participating in the survey. This index theoretically varies from 0 to 1. According to Sharma et al. [23], when relative frequency of citation is 0, it means that nobody refers to the plant/animal as useful, and when relative frequency of citation is 1, it means that all informants in the survey refer to the plant/animal as useful.

#### 2.4.2. Cultural Importance Index

Culturally important species as medicines are identified by the cultural importance index (CII) [24]. The CII was used to determine the most culturally important plant/animal species in each religious group. It can be calculated by the following formula:

(1) $$\begin{array}{c} CII=\overset{u_{nc}}{\underset{u=u_{1}}{\sum }}\;\overset{i_{n}}{\underset{i=i_{1}}{\sum }}\frac{{UR}_{ui}}{N}, \end{array}$$

where NC is the total number of different illness categories (of each *i* species), UR is the total number of use reports for each species, and *N* is the total number of informants in each religious group. The cultural importance index is the sum of the proportion of informants that mention each of the use-categories for a given species. The maximum value of the index equals the total number of different use-categories (NC), which would occur if all informants in a religious group would mention the use of a species in all use-categories. This index was used to estimate the cultural significance of each plant/animal species [24] and to determine to what extent each plant/animal species is present in the memory of the informants belonging to each religious group.

#### 2.4.3. Jaccard Similarity Index

The Jaccard similarity index adapted from Güzel et al. [25] was used to determine the degree of similarity of medicinal plants' use among the different religious groups. The Jaccard similarity index is calculated as follows: Jaccard similarity index = *C* × 100/*A* + *B* − *C*, where *A* is the number of plant species reported by religious group A, *B* is the number of plant species reported by the religious group B, and *C* is the number of plant species reported by both A and B [25].

## 3. Results and Discussion

### 3.1. Demographic Profile of the Participants

The demographic characteristics of the participants were determined and documented through face-to-face interviews using semistructured questionnaire (Tables 1 and 2). A total of 100 randomly selected informants (38 males and 62 females) were interviewed as summarized in Tables 1 and 2. Our finding resembled the study of Ishola et al. [26] where the majority of traditional medicine users were female since they were typically in charge of preparing herbal preparations in the domestic setting. According to Hardy [27], women are the main source of conservation and dissemination of traditional knowledge. Ethnographic investigations revealed that the greatest contribution in terms of traditional information was provided by interviewees belonging to the age group 60–69 years old (*N* = 41). They were followed by informants belonging to the age category 50–59 years old (*N* = 27). This information implies that the young generation neglects traditional medicine practice which might lead to the rapid loss of valuable traditional knowledge regarding the use of medicinal plants [28]. There exist several reasons which might account for the loss of traditional knowledge in the study area: (1) holders of empirical knowledge have died before passing on this knowledge to the younger generation, (2) the younger generation believes more in the efficacy of allopathic medicine, and (3) given the free cost of healthcare facilities provided by the Mauritian government in public hospitals, allopathic medicine is more accessible to the population.

Moreover, the results revealed that the majority of the participants studied till the primary level only (*N* = 64). Our finding is in accordance with the work of Gakuya et al. [29] where elder people with little formal education possess more knowledge concerning the use of medicinal plants. It was also noted that the majority of the informants were retired (*N* = 36) and had a monthly household income of Rs 5001–10000 (1 US$ ≈ Rs 36.00) (*N* = 48). The retirement age in Mauritius is 60 years and above. Nonetheless, some of the participants were found to continue working though they reached the retirement age. The traditional health practitioners (*N* = 5) were found to play vital roles in the study area whereby the indigenous communities rely on them for the provision of herbal medicines. The traditional health practitioners were found to be key custodians of traditional information on the medicinal use of plant and animal species. Their practice of healing involved panoply of methodologies which are considered trustworthy among the indigenous community in the study area. Traditional health practitioners in Mauritius were willing to share their valuable traditional knowledge in order to prevent extinction of this cultural heritage.

In order to allow better comparison of medicinal plants and animals use among the four religious groups present in the study area, the number of participants surveyed in each religious group was approximately equal: 27, 24, 26, and 23 for the Hindu, Muslim, Christian, and Buddhist religious group, respectively. The most common diabetes related complications reported by the informants were hypertension (*N* = 36) followed by high level of cholesterol (27), neuropathic pain (*N* = 25), and cardiovascular diseases (*N* = 12). According to the American Diabetes Association, in type 2 diabetes, hypertension is often present as part of the metabolic syndrome of insulin resistance, while in type 1 diabetes, hypertension may reflect the onset of diabetic nephropathy [30].

### 3.2. Herbal Remedies Used to Manage Diabetes and Related Complications

The present research revealed the ethnobotanical use of 52 plant species belonging to 33 families used to manage diabetes and related complications. Information on medicinal plants obtained from the four religious groups, namely, Hindu, Muslim, Christian, and Buddhist, was arranged alphabetically according to their botanical families along with their ethnomedicinal uses (Table 3).

### 3.3. Source of Medicinal Plants

Informants obtained the medicinal plants from three main sources: gathering from the wild (39%), harvesting from home gardens (37%), and purchasing from the herbalists' store (24%). Our result is in agreement with the work of Singh et al. [31] where the majority of medicinal plants used in the preparation of herbal remedies are obtained from the wild. Indigenous people also cultivate medicinal plants in home garden where medicinal plants are grown in small areas surrounding the house. Moreover, medicinal plants are also grown in clay pots. One informant reported that effective medicinal plants are cultivated close to the house to allow easy accessibility. On the other hand, medicinal plants which are considered rare by the informants and which are not easily available are purchased from the herbalists' store.

### 3.4. Forms of Medicinal Plants

It was found that the informants showed no particular preference for using either fresh or dried plants in the preparation of herbal remedies. They reported that the use of either fresh or dried plants in herbal recipes did not make any difference in the efficacy of the herbal remedies. However, the traditional healers reported that they preferred dried plants which should be kept in open air and not in closed container. Furthermore, drying enabled indigenous people to use medicinal plants during off season. This is supported by the work of Tahraoui et al. [32] whereby plant parts are dried in shade and stored in a house room free of humidity and sunlight for their use during unavailability. Similarly, Lingaraju et al. [33] reported that, in the absence of fresh materials, the dried ones were prescribed in the preparation of herbal remedies. Previous studies had shown that there were quantitative and qualitative differences in the essential oil contents of fresh and dry plant materials [34, 35]. Ishola et al. [26] reported that dry plant materials might not be as potent as freshly collected herbs since some of their enzymes may have been denatured or the heat labile compounds could have been destroyed.

### 3.5. Parts of Medicinal Plants Used in the Preparation of Herbal Remedies

**Figure 3:**
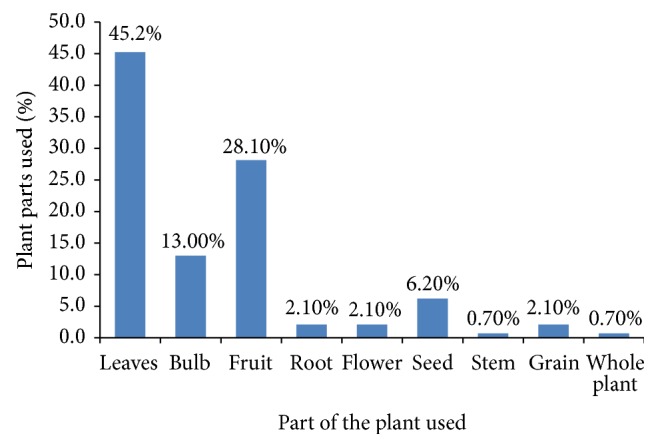
Plant parts employed in herbal remedies by the participants.

In the current investigation, different parts of medicinal plants were documented in the preparation of indigenous herbal medicines to manage diabetes and related complications. Whole plant in addition to different parts of the same plant including leaf, bulb, fruit, root, flower, seed, stem, and grain was used in the preparation of herbal remedies for the management of diabetes and related complications (Figure 3). Leaf was the most frequently used plant parts (45.2%), followed by fruit (28.1%), bulb (13%), seed (6.2%), root (2.1%), flower (2.1%), grain (2.1%), stem (0.7%), and whole plant (0.7%). These observations resonate with finding obtained by Sadeghia and Mahmood [36] in which the part of the plant most commonly used was leaves. According to Tuttolomondo et al. [37], greater accessibility of the aboveground parts of the plants in natural ecosystems and the greater abundance of leaves compared to other plant parts may explain the higher use-frequency of these plant parts in traditional medicine. Leaves are the most favored parts in the preparation of herbal medicines since they contain a high concentration of pharmacologically active secondary metabolites which are valuable in phytotherapy [38, 39]. The result of the present study showed that whole plant is not commonly used in the preparation of herbal remedies because its removal will threaten the conservation of the plant species and hence impair sustainability of indigenous flora in the study area. The result of the study deviates from the work of Cheikhyoussef et al. [40] who observed that roots are mostly used in the preparation of herbal remedies. From the current study, the root of* Rhizophora mucronata*, an endemic plant, was reported to be used against type 2 diabetes. According to Flatie et al. [41], roots contain high concentration of bioactive substances. Nonetheless, frequent harvesting of roots has a negative influence on the survival of the plant species and is therefore discouraged. Different parts of a plant species may contain different types and concentrations of pharmacologically active constituents resulting in distinct pharmacological activities. In the present work, the fruit of* Cucurbita maxima* was reported to be used against type 2 diabetes, and in wound healing, its leaves were used against cataract while its seeds were used against renal failure. The phytochemical analysis of an ethanolic extract of* Cucurbita maxima *seeds revealed the presence of tannins, carbohydrates, glycosides, alkaloids, volatile oils, saponins, proteins, and flavonoids [42].

### 3.6. Method of Preparation of Herbal Remedies

**Figure 4:**
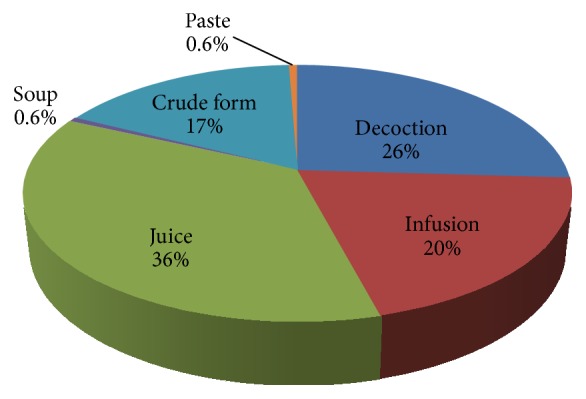
Forms of herbal preparations.

Various preparation modes of herbal medicines like juice, decoction, infusion, crude form, paste, and soup were used by the indigenous community in Mauritius (Figure 4). The most common modes of preparation were juice (36%) followed by decoction (26%) and infusion (20%). Similar finding was reported by Malla et al. [43] in western Nepal where juice was the most commonly used preparation method for administering medicinal plants. Most of the reported herbal preparations are made with water as dilution media. This finding is in line with previous work [44], where water was mostly used as solvent medium in the preparation of herbal remedies. Decoctions are usually prepared by boiling plant parts in water until the amount of water is reduced to half its original amount. According to a study conducted by Zhang et al. [45], on heating various biological reactions are accelerated resulting in many active compounds, hence accounting for the effectiveness of herbal remedies prepared by decoction.

### 3.7. Administration of Herbal Remedies

Regarding the means of administration, oral ingestion (87.1%) was the preferred mode of administration of herbal remedies followed by external use (12.9%). This is in agreement with the finding of Sadeghia and Mahmood [36] where herbal remedies are mostly administered orally. It was reported that the predominance of oral route for administration of herbal remedies can be attributed to the ease of administration without using costly and complex accessories [46].

### 3.8. Botanical Families

**Figure 5:**
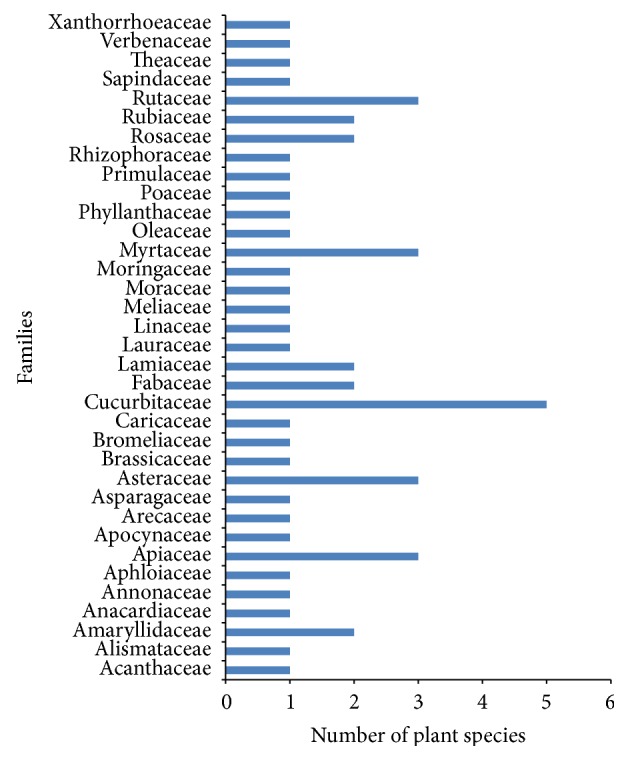
Representative botanical families.

The predominantly quoted medicinal plant families were Cucurbitaceae with five species, followed by Apiaceae, Asteraceae, Myrtaceae, and Rutaceae with three species each and Amaryllidaceae, Fabaceae, Lamiaceae, Rosaceae, and Rubiaceae with two species each. The remaining 23 families were each represented by one species (Figure 5). The Cucurbitaceae family encompasses 800 species distributed mainly in tropical and subtropical regions of the world [47]. The most plausible reason for the predominance of the Cucurbitaceae family in the study area could be due to the large group of plant species belonging to this family which are medicinally valuable due to their phytochemical profile. Moreover, the high citations of the Cucurbitaceae family may be because of the high availability of plant species belonging to this family in the study area. Further, plants belonging to the Cucurbitaceae family contain a group of active secondary metabolites, namely, triterpenoid, which are well known for their bitterness [47], hence justifying their use in the management of diabetes in the present study. It was noted from the current investigation that some of the informants believed that type 2 diabetes is caused by excess sugar in the blood; hence, bitter plants are used to neutralise the excess sugar. In an ethnopharmacological survey conducted in Congo by Katemo et al. [48], it was reported that bitter plants are prescribed to control blood sugar level. Some of the bitter plants recorded from the present study used to manage diabetes with high relative frequency of citation include* Aloe vera *(0.53),* Phyllanthus emblica* (0.52),* Azadirachta indica* (0.46), and* Momordica charantia *(0.46).* Phyllanthus emblica* has been shown to contain an array of bioactive components like quercetin, phyllaemblic compounds, gallic acid, tannins, flavonoids, pectin, vitamin C, terpenoids, and alkaloids which possesses useful biological activities [49–51]. According to Walia and Boolchandani [52],* Phyllanthus emblica* contain high vitamin C content which is effective in controlling diabetes and tannins which has the capacity to enhance glucose uptake and inhibit adipogenesis. The majority of the informants (92%) responded that after consumption of the herbal remedy they felt an improvement in their health state.

### 3.9. Relative Frequency of Citation

Relative frequency of citation was calculated to ascertain the most commonly occurring medicinal plants used for the management of diabetes and related complications and thus aids in the selection of plants for further phytochemical and pharmacological studies.* Citrus aurantifolia *(0.55) was the predominant plant species which exhibited the highest relative frequency of citation demonstrating its importance in indigenous phytotherapy. It is followed by* Morinda citrifolia *(0.54),* Aloe vera *(0.53),* Phyllanthus emblica* (0.52), and* Syzygium cumini* (0.49). Plant species with high relative frequency of citation reflected their popularity due to their strong healing power and they were easily available and affordable in the study area. According to Kpodar et al. [46], other reasons why plant species are cited frequently might be (1) the trust that the indigenous community have in these plants as medicine and (2) the relatively high cost of synthetic drugs. Based on these results, such plants should be focused on for the investigation of bioactive phytochemical constituents and other pharmacological activities. It is important to note that the plants with high relative frequency of citation have been previously screened for their pharmacological activities. Unripen juices of* Citrus aurantifolia* showed antioxidant activities* in vitro* [53]. Moorthy and Reddy [54] reported that the ethanolic extract of the roots of* Morinda citrifolia* lowered blood pressure in an anesthetized dog. An experimental investigation carried out by Alam et al. [55] demonstrated that leaves of* Syzygium cumini *contain the bioactive compounds lupeol, 12-oleanen-3-ol-3*β*-acetate, stigmasterol, and *β*-sitosterol which possess potential antidiabetic activities, hence supporting the traditional use of the leaves for treating diabetes. Some of the plant species reported, namely,* Lysimachia christinae* (0.05),* Prunella vulgaris* (0.09), and* Aloysia citriodora* (0.09), scored low relative frequency of citation since they have been reported by few informants only. Low relative frequency of citation values of these plants imply that traditional knowledge about their use is on the verge of extinction. Furthermore, they were found to be scarce in the study area due to deforestation and urbanization. Since* Lysimachia christinae* is not native to Mauritius, many informants were unaware of this medicinal plant. The traditional Chinese medicine practitioner reported that this plant is imported in its dried form from China.* Lysimachia christinae *contains flavonoid and phenolic compounds which possess promising pharmacological activities* in vivo* [56].

### 3.10. Cultural Importance Index

The cultural importance index showed that* Ocimum tenuiflorum* (0.39),* Cardiospermum halicacabum* (0.09),* Camellia sinensis* (0.27), and* Ophiopogon japonicas* (0.11) are the most culturally important plant species among the Hindu, Muslim, Christian, and Buddhist community, respectively (Table 4). The high cultural importance index of these plants indicates their importance in their respective culture because of their medicinal properties and versatility. These plant species have been used since time immemorial and the medicinal knowledge of these plants has been transmitted from one generation to the next within the specific religious group. According to Tardío and Pardo-de-Santayana [57], the cultural importance index is an efficient tool for highlighting those species with a high agreement for the culture of the study area and hence recognises the shared knowledge of these people. From Table 4, it is evident that plant species which scored very low cultural importance index value in a particular religious group imply that little cultural importance is given to these plant species in traditional medicine in that particular religious group. Tuttolomondo et al. [37] reported that plants with low cultural importance index value indicate that the local populations had little trust in them concerning their use in the treatment of certain pathologies or indicate a fall in traditional plant knowledge regarding medicinal uses of these plants which is an evidence of an ongoing process of cultural erosion. Cultural and religious preferences also influence the use of medicinal plants [58]. Some of the documented medicinal plants were found to play important roles in religious ceremonies among the Hindu community.* Ocimum sanctum *is considered as sacred by the Hindu community. The holy basil worship is done every morning in every Hindu community household in order to keep the family members healthy. Moreover,* Azadirachta indica *and* Mangifera indica *were reported to play fundamental role in “Durga pooja,” a prayer dedicated to goddess “Durga.”

### 3.11. Jaccard Similarity Index

In the current investigation, the Christian community provided us with the highest number of medicinal plants (51) followed by the Hindu (48), Muslim (46), and the Buddhist (37) community. As depicted in Table 5, the Hindu community and the Muslim community showed the highest similarity of medicinal plants usage with Jaccard similarity index value of 95.8. The Hindu and Muslim community are both descendants of Indian indentured labourers who were recruited by the British Empire to work on sugar cane, banana, tea, and coffee plantations. They came from the same village in eastern Uttar Pradesh and western Bihar in northern India and arrived to Mauritius in the same ships [59]. It was observed that the Hindu and Muslim community in Mauritius commonly spoke the “Bhojpuri” dialect which is an amalgam of Creole and Hindi language. Moreover, certain traditions were found to be similar among these two religious groups. For instance, the use of henna to decorate women's hands during weddings was found to be similar among both religious groups. The high degree of similarity of medicinal plants usage between these two communities implies that there has been an exchange of traditional information between these two cultures on the use of medicinal plant species to manage diabetes and related complications. The Hindu community and the Buddhist community showed the least similarity of medicinal plants usage with Jaccard similarity index value of 63.5. The reason for this least similarity is most likely because the Buddhist community have their own system of healing which is distinct to that of the Hindu community. Moreover, the Buddhist community commonly purchased medicinal plants from herbal stores which are imported from China and some of plants employed by the Buddhist community are unknown by the other three religious communities. Moreover, the Buddhist community was observed to be quite reticent to share their traditional knowledge with people not belonging to their cultural group. According to Güzel et al. [25], detailed anthropological studies should be carried out in order to identify factors affecting ethnomedicinal similarities and differences amongst different cultural groups.

### 3.12. Ailment Categories

The reported ailments were grouped into 9 broad categories of diseases (Table 6). The ailment categories treated by the greatest number of medicinal plants were diabetes with 40 listed plant species, followed by diabetic dyslipidemia and hypertension with 19 plant species each. The reasons for this may be due to high prevalence of diabetes in the study area as reported earlier, hence the need to search for more hypoglycemic plants. The efficacy demonstrated by some of the antidiabetic plants identified in this study has previously been documented in either* in vivo* or* in vitro* studies. Ethanolic leaf extract of* Azadirachta indica* was found to normalize blood glucose level in streptozotocin-induced diabetic rats [60].* Syzygium cumini* bark extract lowered blood glucose level in streptozotocin-induced diabetic albino Wistar rats [61]. Aqueous leaf extract of* Graptophyllum pictum* was found to have hypoglycemic effect which is comparable to metformin in alloxan-induced diabetic Wistar rats [62]. Aqueous alcohol extract of the aerial parts of* Bidens pilosa *lowered blood glucose in db/db mice, a type 2 diabetes mouse model [63]. However, herbal medicinal practices may vary among different groups of people in different parts of the world. For example,* Trigonella foenum-graecum* was reported to be used against diabetes, high level of cholesterol, and erectile dysfunction in Mauritius but in Iran it is used against gynaecological problems [36]. The result revealed that 63.5% of the plant species enlisted were employed for the management of more than one kind of disease. This finding is in agreement with previous result described by Yousuf et al. [64] and Gupta et al. [65] where most plant species used by indigenous people have multiple uses.

### 3.13. Cross-Cultural Comparison of Medicinal Plants among the Different Religious Groups

**Figure 6:**
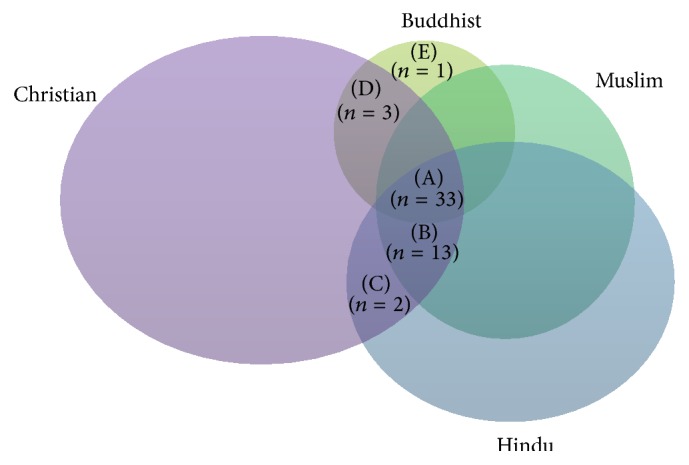
Venn diagram representing the overlap of plant species cited by participants from Hindu, Muslim, Christian, and Chinese communities in Mauritius. (A) Plant species common to Hindu, Muslim, Christian, and Buddhist religious group (*Allium cepa, Allium sativum, Mangifera indica, Apium graveolens, Petroselinum crispum, Catharanthus roseus, Bidens pilosa, Cynara cardunculus, Brassica oleracea, Carica papaya, Cucumis sativus, Cucurbita maxima, Lagenaria siceraria, Momordica charantia, Tamarindus indica, Trigonella foenum-graecum, Ocimum tenuiflorum, Linum usitatissimum, Azadirachta indica, Artocarpus heterophyllus, Psidium guajava, Syzygium cumini, Olea europaea, Phyllanthus emblica, Avena sativa, Rubus alceifolius, Morinda citrifolia, Vangueria madagascariensis, Citrus aurantifolia, Citrus maxima, Murraya koenigii, Camellia sinensis, *and* Aloe vera*). (B) Plant species common to Hindu, Muslim, and Christian religious group only (*Graptophyllum pictum, Annona muricata, Aphloia theiformis, Coriandrum sativum, Cocos nucifera, Sigesbeckia orientalis, Ananas comosus, Luffa acutangula, Persea americana, Moringa oleifera, Rhizophora mucronata, Crataegus laevigata, *and* Cardiospermum halicacabum*). (C) Plant species common to Hindu and Christian religious group only (*Eucalyptus globules, Aloysia citriodora*). (D) Plant species common to Christian and Buddhist religious group only (*Alisma plantago-aquatica, Ophiopogon japonicas, *and* Prunella vulgaris*). (E) Plant species common to Buddhist religious group only (*Lysimachia christinae*).

Though the four religious groups in Mauritius possess different cultures and traditions, it was observed that they have common knowledge about the majority of the reported medicinal plant species. Thirty-three plants species were used commonly among the four religious groups, whereas 13 plant species were common among the Hindu, Muslim, and Christian religious groups only, 2 plants were common between Hindu and Christian communities only, 3 plants were common between the Christian and Buddhist communities only, and 1 plant was used exclusively by the Buddhist community (Figure 6). A high correspondence between the uses of the same medicinal plant species among the four religious groups was surprising. The possible reason accounting for the high similarity of plant species used to manage diabetes and related complications among the four religious groups might be due to the frequent cross-cultural exchange of traditional knowledge on medicinal plants between them to manage these ailments. Moreover, the four religious groups live in close proximity to each other and share similar flora. Lingaraju et al. [33] reported that different ethnic groups influence each other in the adoption and usage of certain medicinal plant species. According to Masevhe et al. [66], the use of plant species by different cultural groups may also indicate their potential pharmacological efficacy. Medicinal plants are not selected at random but exhibit a considerable degree of patterning within one culture [16]. Moreover, plants are selected and used in a consistent manner because of their culturally perceived effectiveness [67, 68]. According to Heinrich et al. [16], the parallel use of plant taxa among different ethnic groups may be due to (1) coincidence (a random selection of similar species), (2) similar criteria for selecting plants, and (3) shared information on the potential usefulness of a plant. Therefore, medicinal plant species which are used in parallel among the four religious groups require further pharmacological, toxicological, and phytochemical analysis for the discovery of potential novel drugs to manage diabetes and related complications.

### 3.14. Animal-Based Remedies Used to Manage Diabetes and Related Complications

In this study, a total of 7 medicinal animal species distributed over 4 classes were recorded for the management of diabetes and related complications (Table 7). Among them, Actinopterygii, Insecta, and Mammalia occupied the most cited classes with two species each. Our present analysis reveals that various parts of animal species were selected as medicinal materials. Whole animal (71.4%) was mostly recorded in the preparation of animal-based remedies followed by honey (14.3%) and skin (14.3%). The result depicts that animal-based remedies were mainly taken in the raw form (57.1%). Our finding is comparable to that of Vijayakumar et al. [69] where medicinal animal species are mostly taken as raw for the treatment of ailments. Based on relative frequency of citation, the most frequently cited medicinal animal species was* Salmo salar* (0.12).* Salmo salar* was found to be commonly used against diabetes in the study area since it contains a polyunsaturated compound, namely, omega-3. Malasanos and Stacpoole [70] reported that omega-3 fatty acids reduce serum lipids and lipoproteins, impair platelet aggregation, increase cell membrane fluidity, and lower blood pressure in diabetic subjects. Further studies are required to confirm the presence of bioactive compounds in these animal remedies reported in the current study. With regard to the administration routes of the animal-based remedies, external application (57.1%) was the most commonly used route of administration. It was observed that certain animal species were used exclusively in a specific religious group. For instance,* Tenrec ecaudatus* was reported to be used against renal failure by the Christian community only. This can be explained by the fact that* Tenrec ecaudatus* is regarded as impure by the Hindu and Muslim community and their religious values forbid them from consuming the meat of this animal. In addition, animal-based remedies were found to be more prominent among the Christian community as compared to the other three religious groups. It is fundamental to carry out studies to evaluate the safety, efficacy, and optimal dosage of the reported animal-based remedies in order to validate their traditional use and ensure proper treatment outcomes.

## 4. Conclusion

To the best of our knowledge, this is the first cross-cultural investigation on traditional therapies used to manage diabetes and related complications in Mauritius. The panoply of information gathered in the present study demonstrates the important recognition of herbal and animal-based remedies among diabetic patients and traditional healers for the management of diabetes and related complications. The high popularity of* Citrus aurantifolia *demonstrates its importance in the study area for the management of diabetes and related complications. However, there is a tendency of using particular types of plants excessively in traditional medicine for its therapeutic effects without concerning its vulnerability to extinction. Hence, appropriate measures should be taken in order to preserve important plant species and emphasis should be placed on the judicious use of medicinal plants. Interviews with individuals from different religious background revealed intra- and interculturally important medicinal plants. Though cultural divergence exists among the 4 religious groups of the island, a high degree of similarity of medicinal plants usage among them has been observed. The possible reason for the high correspondence of the use of the same medicinal plant species is due to the frequent cross-cultural exchange of traditional knowledge on medicinal plants. Nonetheless, the use of certain medicinal plants and animal species has been found to be confined in only a particular religious group. According to Mustafa et al. [71], cross-cultural studies could be important for proposing culturally sensitive ways of using plant natural resources in future sustainable economic development initiatives. Culturally important plant species such as* Ocimum tenuiflorum*,* Cardiospermum halicacabum*,* Camellia sinensis,* and* Ophiopogon japonicas* should be subjected to detailed screenings for pharmacologically active metabolites for the discovery of new therapeutic agents. As a concluding note, the present study reflects the rich cultural heritage in terms of ethnomedicinal knowledge possessed by the different religious groups in Mauritius. However, this knowledge is in jeopardy due to the lack of interest shown by the younger generation. Therefore, we keenly emphasise the importance of transmitting this precious knowledge which is vanishing at an alarming rate in order to safeguard our cultural heritage.

## Supplementary Material

A semi-structured questionnaire was used to seek data from indigenous people. The questionnaire comprised of both open and closed ended questions ranging from demographic data of the respondents to the traditional remedies (herbal and animal-based) used by the indigenous people.

## Figures and Tables

**Table 1:** Demographic data of the informants (*N* = 100).

Variable	Categories	Frequency (*n* = 100)
Age (years)	30–39	2
	40–49	17
	50–59	27
	60–69	41
	70–79	8
	≥80	5

Sex	Male	38
	Female	62

Level of education	No formal education	7
	Primary	64
	Secondary	20
	Tertiary	9

Occupation	Retired	36
	Nongovernment officer	25
	Housewife	18
	Government officer	16
	Traditional healer	2
	Ayurvedic medicine practitioner	2
	Traditional Chinese medicine practitioner	1

Monthly household income	<Rs 5000	2
	Rs 5001–10000	48
	Rs 10001–20000	40
	Rs 20001–30000	8
	>Rs 30001	2

Religion	Hindu	27
	Muslim	24
	Christian	26
	Buddhist	23

Diabetes related complications	Hypertension	36
	High level of cholesterol	27
	Neuropathic pain	25
	Cardiovascular diseases	12
	Cataracts	9
	Urinary tract infections	7
	Renal failure	5
	Foot ulcers	4
	Gangrene	3
	Infected wounds	3
	Stress	2
	Dry skin	2
	Erectile dysfunction	1
	Hearing loss	1
	Memory loss	1
	Depression	1

**Table 2:** Age and gender distribution within each religious community.

Religious community	Age	Number of participants	Gender	Number of participants
Hindu	30–39	1	Male	12
	40–49	3	Female	15
	50–59	8		
	60–69	7		
	70–79	6		
	≥80	2		

Muslim	30–39	0	Male	11
	40–49	4	Female	13
	50–59	3		
	60–69	14		
	70–79	2		
	≥80	1		

Christian	30–39	1	Male	10
	40–49	4	Female	16
	50–59	7		
	60–69	13		
	70–79	0		
	≥80	1		

Buddhist	30–39	0	Male	5
	40–49	6	Female	18
	50–59	9		
	60–69	7		
	70–79	0		
	≥80	1		

**Table 3:** List of medicinal plants and polyherbal formulations with their related information used against diabetes and related complications reported by the informants.

Family	Scientific name of plant (identification number)	Local name of plant	Indication	Part of plant used	Method of preparation and administration	RFC	CII_H_	CII_M_	CII_C_	CII_B_
Acanthaceae	*Graptophyllum pictum *(L.) Griff. (AM15)	Lait de vierge	Type 2 diabetes	L	Prepare a decoction with 3 leaves and drink 2 cups daily for 1 week.	0.16	0.02	0.04	0.10	0.00

Alismataceae	*Alisma plantago-aquatica* subsp. orientale (Sam) Sam. (AM09)	—	High level of cholesterol	—	Sold as a Chinese tea against cholesterol. Prepare an infusion with the teabags which contain *Alisma orientalis* (*Rhizoma alismatis*), *Radix angelicae sinensis*, *Herba artemisiae capillaris*, Hawthorn berry, *Rhizoma atractylodis macrocephalae, Semen ziziphi spinosae,* and Chinese tea. Drink 1 cup daily.	0.12	0.00	0.00	0.03	0.09

			Type 1 diabetes	B	Prepare a decoction with the bulb and drink 1 cup daily for 1 week.					
			Type 2 diabetes	B	Prepare a decoction with the bulb and drink 1 cup daily for 1 week.					
			Type 2 diabetes	B	Prepare juice with the bulb and add 1 teaspoon of honey. Drink 1 cup daily for 3 months.					
			High level of cholesterol	B	Prepare a decoction with the bulb and drink 1 cup daily for 1 week.					
			Renal failure	B	Prepare a decoction with the bulb and drink 1 cup daily for 1 week.					
	*Allium cepa *L. (AM04)	Zoiyon/oignon	Hearing loss	B	Crush and press the bulb to obtain the juice and mix 30 g of the juice with 30 g of water. Heat and instill 3-4 drops in the affected ear.	0.48	0.28	0.12	0.17	0.03
Amaryllidaceae			Erectile dysfunction	B	Prepare juice with the bulb and add 1 teaspoon of honey. Drink 1 cup daily for 3 months.					
			Cataract	B	Prepare a decoction with the bulb and add 2 teaspoons of honey. Allow it to cool and use it as an eyebath daily.					
			Type 2 diabetes	B	Prepare a decoction with 2-3 cloves and drink 1 cup thrice per week.					
			Cataract	B	Prepare a decoction with 2-3 cloves and drink 1 cup thrice per week.					
			Renal failure	B	Consume 2-3 raw cloves daily for 1 week.					
	*Allium sativum* L. (AM39)	L'ail	Hypertension	B	Swallow 2 small cloves with a cup of water thrice per week.	0.42	0.24	0.09	0.22	0.06
			Wound	B	Crush and press the bulb to obtain juice and apply the juice on the wound daily till healing.					
			Ulcer	B	Crush and press the bulb to obtain juice and apply the juice on the ulcer daily till healing.					

Anacardiaceae	*Mangifera indica *L. (AM20)	Mangue	Type 2 diabetes	L	Prepare an infusion of the leaves and drink 1 cup twice per week.	0.31	0.17	0.03	0.10	0.01

Annonaceae	*Annona muricata *L. (AM29)	Coronsol	Hypertension	L	Prepare an infusion of the leaves and drink 1 cup twice per week.	0.16	0.07	0.03	0.06	0.00

Aphloiaceae	*Aphloia theiformis* ^*∗*^ (Vahl) Benn. (AM51)	Fandamane	Cataract	L	Prepare an infusion with the leaves and wash the eyes with it daily.	0.18	0.08	0.02	0.10	0.00
			Type 2 diabetes	L	Prepare an infusion of the leaves and drink 1 cup twice per week.					

Apiaceae	*Apium graveolens *L. (AM07)	Céleri	Type 2 diabetes	L	Prepare a decoction with the leaves and drink 1 cup twice per week.	0.37	0.12	0.08	0.19	0.03
			Hypertension	L	Prepare a decoction with the leaves and drink 1 cup twice per week.					
	*Coriandrum sativum *L. (AM11)	Cotomili	Type 2 diabetes	L	Prepare an infusion with the leaves and drink 1 cup twice per week.	0.18	0.05	0.02	0.11	0.00
	*Petroselinum crispum *(Mill.) Nyman exA.W. Hill. (AM52)	Persil	High level of cholesterol	L	Prepare a decoction with the leaves and drink 1 cup twice per week.	0.27	0.12	0.07	0.16	0.01
			High level of cholesterol	L	Prepare a soup with the leaves together with *Apium graveolens *L. and *Allium ampeloprasum *var.* porrum*. Consume it twice per week.					
			Type 2 diabetes	L	Prepare a decoction with the leaves and drink 1 cup twice per week.					
			Renal failure	L	Prepare a decoction with the leaves and drink 1 cup twice per week.					
			Hypertension	L	Prepare juice with the leaves together with *Daucus carota* and *Apium graveolens *L. Drink 1 cup twice per week.					

Apocynaceae	*Catharanthus roseus *L. G.Don (AM17)	Saponnaire (blanc)	Type 2 diabetes	L	Prepare an infusion with 7 leaves in 2 cups of hot water. Drink 1 cup thrice per week.	0.18	0.04	0.05	0.08	0.01

Arecaceae	*Cocos nucifera *L. (AM21)	Coco	Cataract	Fr	Instill 2 drops of oil in the eye twice per day.	0.23	0.08	0.02	0.12	0.00
			Type 2 diabetes	Fr	Prepare a decoction with the young fruits and drink 1 cup thrice per week.					
			Renal failure	Fr	Drink 1 cup of the fruit water four times per week.					
			Renal failure	R	Prepare a decoction of the root and drink 1 cup twice per week.					

Asparagaceae	*Ophiopogon japonicus *(Thunb.) Ker Grawl. (AM22)	—	Type 2 diabetes	—	Sold as Chinese antidiabetic tea. Prepare an infusion with the tea bags which contain *Ophiopogon japonicas* (Radix ophiopogonis), fragrant solomonseal rhizome, Chinese yam, Hawthorn berry, *Radix puerariae,* and white tea. Drink 1 cup daily.	0.13	0.00	0.00	0.02	0.11

Asteraceae	*Bidens pilosa *L. (AM31)	Lavilbag	Type 2 diabetes	L	Prepare a decoction of 3 leaves and drink 1 cup twice per week.	0.28	0.14	0.07	0.10	0.04
			Hypertension	L	Prepare a decoction of 3 leaves and drink 1 cup twice per week.					
			Type 2 diabetes	L	Prepare an infusion with 10 g of leaves in 1 L of water and drink 1 cup twice/thrice per week.					
			High level of cholesterol	L	Prepare an infusion of the leaves and drink 1 cup twice per day for 1 week.					
	*Cynara cardunculus *L. (AM24)	Artichaut	High level of cholesterol	L	Prepare juice with the leaves and drink 1 cup daily for 1 week.	0.31	0.10	0.06	0.15	0.07
			Atherosclerosis	L	Prepare an infusion of the leaves and drink 1 cup twice per day for 1 week.					
			Atherosclerosis	L	Prepare juice with the leaves and drink 1 cup twice per day for 1 week.					
	*Sigesbeckia orientalis *L. (AM05)	Herbe de flacq	Type 2 diabetes	L	Prepare a decoction of the leaves and drink 1 cup twice per week.	0.34	0.17	0.09	0.08	0.00
			Type 2 diabetes	L	Prepare a decoction of the leaves together with *Aphloia theiformis*, *Faujasiopsis flexuosa*, *Rubus alceifolius*, *Ravenala madagascariensis,* and* Rhizophora mucronata*. Drink 1 cup twice per week.					

Brassicaceae	*Brassica oleracea *L. (AM33)	Li chou	Cardiovascular disease	L	Prepare juice with the leaves and drink 1 cup daily for 1 week.	0.37	0.13	0.11	0.17	0.02
			Type 2 diabetes	L	Prepare juice with the leaves and drink 1 cup daily for 1 week.					
			Wound	L	Apply the leaves as a cataplasm on the wound.					
			Cataract	L	Crush and press the leaves to obtain juice and instill 3-4 drops in each eye 2 hours daily till healing.					
			Hearing loss	L	Prepare juice with the leaves and mix equal amount of the juice with equal amount of the juice of *Citrus medica *L. fruit. Instill 2 drops in the ears daily before going to bed.					

Bromeliaceae	*Ananas comosus *(L.) Merr. (AM30)	Anana	Renal failure	Fr	Consume ripe fruit twice per week.	0.15	0.03	0.01	0.13	0.00
			Cardiovascular disease	Fr	Prepare juice with the fruit and water and drink 1 cup twice per week.					

Caricaceae	*Carica papaya *L. (AM45)	Papaya	Hypertension	Fr	Consume ripe fruit half an hour before breakfast thrice per week.	0.21	0.07	0.08	0.05	0.02
			High level of cholesterol	Fr	Crush and press the raw fruit to obtain milky liquid and drink 1 teaspoon twice per week.					
			Cardiovascular disease	Fr	Prepare juice with the fruit together with *Daucus carota*. Drink 1 cup thrice per week.					

Cucurbitaceae	*Cucumis sativus *L. (AM10)	Concombre	Type 1 diabetes	Fr	Prepare juice with the fruit and water and drink 1 cup on alternative days.	0.14	0.02	0.01	0.12	0.01
			Type 2 diabetes	Fr	Prepare juice with the fruit and water and drink 1 cup on alternative days.					
	*Cucurbita maxima *Duchesne (AM01)	Giromon	Type 2 diabetes	Fr	Prepare a decoction with the peels in water and drink 1 cup daily for 1 week.	0.23	0.02	0.07	0.14	0.01
			Cataract	Fl	Crush and press the flower to obtain juice. Apply juice as compress externally on the eyes.					
			Renal failure	Se	Seeds are dried in bright sunlight for 1 day and eaten raw the following day. Seeds should be consumed thrice per week.					
			Wound	Fr	Prepare juice with the fruit and apply it on wound till healing.					
	*Lagenaria siceraria *(Molina) Standl. (AM41)	Calebasse	Type 2 diabetes	Fr	Prepare a decoction of the peels in water by allowing it to boil for 20 minutes. Drink 1 cup for 3 days.	0.32	0.14	0.09	0.09	0.07
			High level of cholesterol	L	Prepare a decoction with the leaves and drink 1 cup twice per week.					
			Hypertension	L	Prepare a decoction with the leaves and drink 1 cup twice per week.					
	*Luffa acutangula *(L.) Roxb. (AM08)	Patole	Hypertension	L	Crush and press 3–5 leaves to obtain juice and drink it twice per week.	0.17	0.03	0.05	0.11	0.00
			Cardiovascular disease	L	Prepare juice with the leaves together with *Swertia chirayita* and honey. Drink 1 cup twice per week.					
			Type 2 diabetes	L	Eat 2-3 leaves twice per week.					
			Type 2 diabetes	L	Extract the liquid by crushing the leaves and drink 1 teaspoon twice per week.					
			Type 2 diabetes	Fr	Extract the liquid by crushing the fruit and drink 1-2 teaspoons twice per week.					
			Type 2 diabetes	Se	Dry the seeds in bright sunlight during the day and at night allow them to soak in a cup of water and drink it the next morning on an empty stomach.					
			Type 2 diabetes	L	Prepare juice with 3 leaves and add *Piper nigrum*. Drink it once per week.					
	*Momordica charantia *L. (AM03)	Margose	Type 2 diabetes	Fr	Prepare juice with the fruit together with *Phaseolus vulgaris *L., *Malus domestica, *and *Aloe barbadensis*. Drink 1 cup once per week.	0.46	0.23	0.09	0.11	0.05
			Type 2 diabetes	Fr	Prepare juice with the fruit together with the fruit of *Phyllanthus emblica *and the fruit of *Syzygium cumini*. Drink 1 cup twice per week.					
			Type 2 diabetes	Fr	Prepare juice with the fruit together with *Phaseolus vulgaris *L. and drink 1 cup twice per week.					
			Type 2 diabetes	Fr	Prepare juice with the fruit together with *Cucumis sativus* and drink 1 cup twice per week.					
			High level of cholesterol	Fr	Prepare juice with the fruit together with the fruit of *Phyllanthus emblica *and the fruit of *Syzygium cumini*. Drink 1 cup twice per week.					

Fabaceae	*Tamarindus indica* L. (AM37)	Tamarin	Hypertension	Fr	Prepare juice with the pulp and water and drink 1 cup twice per day for 1 day.	0.39	0.12	0.09	0.18	0.02
			Pain	L	Prepare a foot bath with a decoction of the leaves mixed with 1 teaspoon of salt.					
			Type 2 diabetes	Se	Prepare a decoction with the seeds and drink 1 cup thrice per week.					
	*Trigonella foenum-graecum *L. (AM18)	Methi	High level of cholesterol	Se	Soak the seeds in 1 glass of water for 1 night and drink it the next morning on an empty stomach.	0.43	0.23	0.06	0.22	0.01
			Erectile dysfunction	Se	Prepare a decoction with 1 teaspoon of seeds and 2 cups of water. Drink 1 cup on an empty stomach in the morning daily for 1 week.					

			Type 2 diabetes	L	Crush and press the leaves to obtain juice and drink 2 teaspoons twice per week.					
			Type 2 diabetes	L	Consume 2 raw leaves twice per week.					
			Hypertension	L	Crush and press the leaves to obtain juice and drink 2 teaspoons twice per week.					
			Cataract	L	Crush 2 leaves and press to obtain juice and take 1 drop of the juice in the eye daily.					
	*Ocimum tenuiflorum *L. (AM26)	Tulsi	Cataract	L	Crush and press 3-4 leaves to obtain juice and add 2 teaspoons of honey. Instill 2 drops of the mixture in the eye each night for 5 days.	0.48	0.39	0.15	0.16	0.02
Lamiaceae			Erectile dysfunction	L	Prepare a decoction with 3 leaves together with 3 leaves of *Piper betle* and drink 1 cup twice per week.					
			Wound	L	Crush and press the leaves to obtain juice and mix the juice with the oil of *Cocos nucifera* that has previously been heated and apply it on the wound.					
			High level of cholesterol	L	Crush and press 3 leaves to obtain juice and add 2 teaspoons of honey and drink twice per week.					
	*Prunella vulgaris *L. (AM28)	—	Hypertension	—	Sold as Chinese antihypertensive tea. Prepare an infusion with the tea bags which contain *Prunella vulgaris *L. (Selfheal spike), *Ramulus uncariae cumuncis*, *Fructus leonuri*, and Chinese oolong tea. Drink 1 cup daily.	0.09	0.00	0.00	0.01	0.08

Lauraceae	*Persea americana *Mill. (AM34)	Avocat	Cataract	Fr	Prepare juice with 2 cups of yoghurt, 1/2 a cup of the fruit, and 1/2 a cup of water and drink 1 cup once per week.	0.21	0.08	0.04	0.11	0.00
			High level of cholesterol	Fr	Prepare juice with 2 cups of yoghurt, 1/2 a cup of the fruit, and 1/2 a cup of water and drink 1 cup once per week.					

Linaceae	*Linum usitatissimum *Linnaeus. (AM40)	Grain de lin	Type 2 diabetes	Se	Soak the seeds in a cup of water at night and drink it the next morning on an empty stomach. Drink it thrice per week.	0.34	0.16	0.13	0.08	0.01
			Renal failure	Se	Soak the seeds in a cup of water at night and drink it the next morning on an empty stomach. Drink it thrice per week.					
			High level of cholesterol	Se	Soak the seeds in a cup of water at night and drink it the next morning on an empty stomach. Drink it thrice per week.					

Meliaceae	*Azadirachta indica* A. Juss (AM16)	Neem, lila perche	Type 2 diabetes	L	Prepare a decoction of the leaves and drink 1 cup twice per week.	0.46	0.21	0.12	0.09	0.04
			Type 2 diabetes	L	Crush the leaves and make small balls with them and allow them to dry in the sun. The following day, swallow 2 balls with 1 glass of water twice per week.					

Moraceae	*Artocarpus heterophyllus* Lam. (AM36)	Zack	Type 2 diabetes	Fr	Prepare a decoction with the young fruits and drink 1 cup daily for 1 week.	0.19	0.07	0.02	0.08	0.02

Moringaceae	*Moringa oleifera *Lam. (AM42)	Brède mouroungue	Type 2 diabetes	L	Crush and press the leaves to obtain juice. Mix it with milk and drink 1 cup twice per week.	0.26	0.12	0.06	0.16	0.00
			High level of cholesterol	L	Crush and press the leaves to obtain juice. Mix it with milk and drink 1 cup twice per week.					
			Hypertension	R	Prepare a decoction with the root and drink 1 cup twice per week.					
			Hypertension	St	Prepare a decoction with the stem and drink 1 cup twice per week.					

Myrtaceae	*Eucalyptus globulus *Labill. (AM32)	Eucalyptus	Type 2 diabetes	L	Prepare an infusion with 2-3 leaves and drink 1 cup twice per week.	0.13	0.05	0.00	0.08	0.00
				L	Prepare an infusion with 3 leaves and drink 1 cup daily for 1 week.					
	*Psidium guajava *L. (AM50)	Goyave	Type 2 diabetes	Fr	Consume rip fruit thrice per week.	0.38	0.17	0.04	0.15	0.02
				Fr	Prepare a juice of the fruit and drink 1 cup daily for 1 week.					
	*Syzygium cumini *L. Skeels (AM06)	Jamblon	Type 2	L	Prepare a decoction with the leaves and drink 1 cup daily for 1 week.	0.49	0.19	0.14	0.13	0.03
				Fr	Consume 10 ripe fruits thrice per week.					
				Fr	Prepare juice with 1 cup of the fruits and 2 cups of water. Drink 1 cup twice per week.					
				Se	Prepare a decoction with the seeds and drink 1 cup twice per week.					
				Fr	Sold as an Ayurvedic preparation known as “Karela jamun” which contains *Syzygium cumini *and *Momordica charantia.* Drink 5–10 ml of the preparation with 1/2 a glass of water twice per day.					
				Fr	Sold as an Ayurvedic preparation known as “Yesaka” which contains * Phyllanthus emblica*, *Terminalia chebula*, *Terminalia bellerica, Syzygium cumini*, *Picrorhiza kurroa, Swertia chirata*, *Tinospora cordifolia, Gymnema sylvestre*, *Momordica charantia*, *Curauma longa, Salacia chinensis* Linn., and* Melia azadirachta. *Drink 1 tablespoon twice per day.					

Oleaceae	*Olea europaea *L. (AM02)	Zolive	Hypertension	L	Prepare an infusion of the leaves and drink 1 cup daily for 1 week.	0.24	0.10	0.04	0.13	0.02
			Cardiovascular disease	L	Prepare an infusion of the leaves and drink 1 cup daily for 1 week.					
			Type 2 diabetes	L	Prepare an infusion of the leaves and drink 1 cup daily for 1 week.					

Phyllanthaceae	*Phyllanthus emblica *L. (AM13)	Amla	Type 2 diabetes	—	Sold as an Ayurvedic preparation called “Triphala” containing *Phyllanthus emblica*, *Bellirica myrobalan, *and *Chebulic myrobalan*. Drink 1 tablespoon daily.	0.52	0.25	0.14	0.16	0.06
			Type 2 diabetes	—	Sold as an Ayurvedic preparation known as “Amla karela” which contains *Phyllanthus emblica* and *Momordica charantia.* Drink 10–30 ml of the preparation daily in 100 ml of water.					
			Type 2 diabetes	Fr	Consume raw fruits thrice per week.					
			Type 2 diabetes	Fr	Prepare juice with 1 cup of fruits and 1 cup of water and add 1 teaspoon of honey to the juice (optional). Drink 1 cup thrice per week.					
			High level of cholesterol	Fr	Prepare juice with 1 cup of fruits and 1 cup of water and drink 1 cup thrice per week.					

Poaceae	*Avena sativa *L. (AM35)	Oatmeal	Type 2 diabetes	Gr	The grains are soaked in 1 cup of water during the night and drunk in the morning on an empty stomach.	0.32	0.19	0.08	0.13	0.02
			High level of cholesterol	Gr	The grains are soaked in 1 cup of water during the night and drunk in the morning on an empty stomach.					
			Dry skin	Gr	The grains are crushed into fined powders and mixed with 1 tablespoon of almond oil to form a paste and applied on wet skin after bath. Leave it for 10 minutes then rinse it with water.					

Primulaceae	*Lysimachia christinae *Hance (AM19)	—	Urinary tract infection	L	Prepare a decoction of the leaves and drink 1 cup daily for 1 week.	0.05	0.00	0.00	0.00	0.05

Rhizophoraceae	*Rhizophora mucronata* ^*∗*^ Lam. (AM25)	Manglier	Type 2 diabetes	R	Prepare an infusion of the roots and drink 1 cup twice per week.	0.39	0.16	0.04	0.19	0.00

Rosaceae	*Crataegus laevigata *Poir. DC. (AM38)	Aubépine	Cataract	L	Prepare an infusion of the leaves and wash the eye with it.	0.32	0.14	0.06	0.17	0.00
			High level of cholesterol	L	Prepare an infusion of the leaves and drink 1 cup twice per week.					
			Hypertension	Fl	Prepare an infusion with 1 teaspoon of flower and drink 2 cups per day twice per week.					
			Atherosclerosis	Fl	Prepare an infusion with 1 teaspoon of flower and drink 2 cups per day twice per week.					
	*Rubus alceifolius *Poir. (AM14)	Piquant loulou	Type 2 diabetes	L	Prepare a decoction with the leaves and drink 1 cup twice per week.	0.36	0.11	0.09	0.14	0.02

Rubiaceae	*Morinda citrifolia *L. (AM12)	Noni	Type 2 diabetes	Fr	The fruit is peeled, crushed, and pressed to obtain the juice. Drink 1 cup thrice per week.	0.54	0.24	0.19	0.25	0.07
			High level of cholesterol	Fr	The fruit is peeled, crushed, and pressed to obtain the juice. Drink 1 cup thrice per week.					
			Hypertension	L	Prepare an infusion with the leaves and drink 1 cup twice per week.					
			Pain	L	Apply warm oil on the painful area and bind it with the leaves.					
	*Vangueria madagascariensis *J.F.Gmel. (AM27)	Vavangue	Type 2 diabetes	L	Prepare a decoction of the leaves and drink 1 cup twice per week.	0.37	0.12	0.08	0.19	0.02
			Hypertension	L	Prepare a decoction of the leaves and drink 1 cup twice per week.					

Rutaceae			Hypertension	Fr	Peel and press the fruit to obtain the juice and drink 1 cup.					
			Type 2 diabetes	Fr	Prepare juice with the fruit together with 1 clove of *Allium sativum*, 1 teaspoon of honey, and 1 cup of water. Drink 1 cup twice per week.					
			Renal failure	L	Peel and press the fruit to obtain juice and drink 1 cup twice per week.					
	*Citrus aurantifolia *(Christm.) Swingle (AM49)	Limon	Renal failure	Fr	Peel and prepare juice with the pulp and add 1 teaspoon of honey. Drink thrice per week in the morning.	0.55	0.24	0.10	0.23	0.05
			Cardiovascular disease	L	Prepare an infusion with 4 leaves and drink 1 cup thrice per week.					
			Cardiovascular disease	Fr	Prepare juice with the fruit together with 1 clove of *Allium sativum *L., 1 teaspoon of honey, and 1 cup of water. Drink 1 cup twice per week.					
			Cataract	Fr	Prepare juice with the pulp and add 2 teaspoons of honey and use it as an eyebath daily.					
			Type 2 diabetes	Fr	Prepare a decoction of the peels in water and drink 1 cup thrice per week.					
	*Citrus maxima *(Burm.) Osbeck (AM47)	Pamplemousse	High level of cholesterol	Fr	Prepare a decoction of the peels in water and drink 1 cup thrice per week.	0.48	0.20	0.18	0.19	0.02
			High level of cholesterol	Fr	Prepare juice with the fruit together with *Daucus carota* and 2 cm of *Zingiber officinale *root. Drink 1 cup once per week.					
	*Murraya koenigii *(L.) Spreng (AM44)	Carripoulet	Hypertension	L	Prepare an infusion with 3 leaves and drink 1 cup twice per week.	0.18	0.09	0.03	0.05	0.01

Sapindaceae	*Cardiospermum halicacabum *L. (AM23)	Pocpoc	Gangrene	L	Prepare an infusion of the leaves together with *Senna alexandrina* Mill. and *Senna alata* L. Drink 1 cup twice per week.	0.21	0.08	0.09	0.05	0.00
			Wound	L	Crush the leaves and apply them on the wound as a poultice.					
			Type 2 diabetes	L	Prepare a decoction of the leaves and drink 1 cup twice per week.					

Theaceae	*Camellia sinensis *L. Kuntze (AM48)	Thé vert	Cataract	L	Prepare an infusion with the tea bags and wash the eye with it.	0.45	0.26	0.12	0.27	0.08
			Type 2 diabetes	L	Prepare an infusion with the tea bags and drink 1 cup twice per week.					
			Hypertension	L	Prepare an infusion with the tea bags and drink 1 cup twice per week.					
			High level of cholesterol	L	Prepare an infusion with the tea bags and drink 1 cup twice per week.					
			High level of cholesterol	L	Prepare an infusion with the teabags together with *Cinnamomum verum.* Drink 1 cup twice per week at night.					

Verbenaceae	*Aloysia citriodora *Palau (AM45)	Verveine	Cardiovascular disease	W	Prepare an infusion with 1 teaspoon of the plant in 1 cup of hot water. Allow it to infuse for 10 minutes and drink 1 cup thrice per week.	0.09	0.02	0.00	0.07	0.00

Xanthorrhoeaceae	*Aloe vera* (L.) Burm.f. (AM46)	Aloe vera	Type 2 diabetes	L	Gel is removed from the leaf pulp and 2 tablespoons are eaten daily in the morning for 1 week.	0.53	0.17	0.15	0.23	0.06
			Type 2 diabetes	L	Sold as an Ayurvedic juice. 10 ml taken twice daily after meal.					
			High level of cholesterol	L	Prepare a mixture with 2 tablespoons of the gel removed from the leaf pulp, 1 cup of yoghurt, and 1/2 a cup of water. Mix all in a juicer and drink 1 cup of the juice obtained twice per week.					
			Gangrene	L	Prepare a footbath with the decoction of the leaf mixed with 1 teaspoon of salt and 1 teaspoon of vinegar. Soak foot for 30–45 minutes daily for 1 week.					

RFC: relative frequency of citation, CII_H_: cultural importance index among the Hindu community, CII_M_: cultural importance index among the Muslim community, CII_C_: cultural importance index among the Christian community, and CII_B_: cultural importance index among the Buddhist community. *Plant part used*: R, root; L, leaf; Fr, fruit; Se, seeds; W, whole plant; B, bulb; St, stem; Fl, flower; Gr, grain. ^*∗*^List of endemic plants.

**Table 4:** Culturally most important plant and animal species used against diabetes and related complications.

Religious groups	Hindu	Muslim	Christian	Buddhist
Plant species	*Ocimum tenuiflorum* (0.39)	*Cardiospermum halicacabum* (0.09)	*Camellia sinensis* (0.27)	*Ophiopogon japonicas* (0.11)
	*Allium cepa* (0.28)	*Carica papaya* (0.08)	*Morinda citrifolia* (0.25)	*Alisma plantago-aquatica* (0.09)
	*Phyllanthus emblica* (0.25)		*Aloe vera* (0.23)	*Prunella vulgaris* (0.08)
	*Allium sativum* (0.24)		*Apium graveolens* (0.19)	*Lysimachia christinae* (0.05)
	*Citrus aurantifolia* (0.24)		*Rhizophora mucronata* (0.19)	
	*Momordica charantia* (0.23)		*Vangueria madagascariensis* (0.19)	
	*Trigonella foenum-graecum* (0.23)		*Tamarindus indica* (0.18)	
	*Azadirachta indica* (0.21)		*Brassica oleracea* (0.17)	
	*Citrus maxima* (0.20)		*Crataegus laevigata* (0.17)	
	*Avena sativa* (0.19)		*Moringa oleifera* (0.16)	
	*Syzygium cumini* (0.19)		*Petroselinum crispum* (0.16)	
	*Mangifera indica* (0.17)		*Cynara cardunculus* (0.15)	
	*Psidium guajava* (0.17)		*Cucurbita maxima* (0.14)	
	*Sigesbeckia orientalis* (0.17)		*Rubus alceifolius* (0.14)	
	*Linum usitatissimum* (0.16)		*Ananas comosus* (0.13)	
	*Bidens pilosa* (0.14)		*Olea europaea* (0.13)	
	*Lagenaria siceraria* (0.14)		*Cocos nucifera* (0.12)	
	*Murraya koenigii* (0.09)		*Cucumis sativus* (0.12)	
	*Annona muricata* (0.07)		*Coriandrum sativum* (0.11)	
			*Luffa acutangula* (0.11)	
			*Persea americana* (0.11)	
			*Aphloia theiformis* (0.10)	
			*Graptophyllum pictum* (0.10)	
			*Artocarpus heterophyllus* (0.08)	
			*Catharanthus roseus* (0.08)	
			*Eucalyptus globules* (0.08)	
			*Aloysia citriodora* (0.07)	

Animal species	*Anguilla japonica* (0.02)		*Salmo salar* (0.08)	*Rattus rattus* (0.02)
			*Apis mellifera* (0.03)	
			*Tenrec ecaudatus* (0.03)	
			*Helix aspersa* (0.02)	
			*Periplaneta americana* (0.02)	

**Table 5:** Jaccard similarity index for the different religious groups of Mauritius regarding the number of medicinal plants used to manage diabetes and related complications.

	Hindu	Muslim	Christian	Buddhist
Hindu	—	95.8	94.1	63.5
Muslim	95.8	—	90.2	66.0
Christian	94.1	90.2	—	69.2
Buddhist	63.5	66.0	69.2	—

**Table 6:** The use of plant-based remedies and animal-based remedies by illness categories.

Illness categories	Ethnomedicinal applications	Plant species	Animal species
Diabetic angiopathy	Atherosclerosis, cardiovascular disease	*Cynara cardunculus, Brassica oleracea, Ananas comosus, Carica papaya, Luffa acutangula, Olea europaea, Crataegus laevigata, Citrus aurantifolia, *and* Aloysia citriodora*	—

Diabetic nephropathy	Renal failure	*Allium cepa, Allium sativum, Petroselinum crispum, Cocos nucifera, Ananas comosus, Cucurbita maxima, Linum usitatissimum, *and* Citrus aurantifolia*	*Tenrec ecaudatus*

Diabetic neuropathy	Pain, erectile dysfunction, and hearing loss	*Allium cepa, Brassica oleracea, Tamarindus indica, Trigonella foenum-graecum, Ocimum tenuiflorum, *and* Morinda citrifolia*	*Anguilla japonica*

Eye diseases	Cataracts	*Allium cepa, Allium sativum, Aphloia theiformis,Cocos nucifera, Brassica oleracea, Cucurbita maxima, Ocimum tenuiflorum, Persea americana, Crataegus laevigata, Citrus aurantifolia, *and* Camellia sinensis*	*Helix aspersa* *Apis mellifera*

Diabetic dyslipidemia	High level of cholesterol	*Alisma plantago-aquatica, Allium cepa, Petroselinum crispum, Cynara cardunculus,* *Carica papaya, Lagenaria siceraria, Momordica charantia, Trigonella foenum-graecum, Ocimum tenuiflorum, Persea americana, Linum usitatissimum, Moringa oleifera, Phyllanthus emblica, Avena sativa, Crataegus laevigata*, *Morinda citrifolia, Citrus maxima, Camellia sinensis, *and* Aloe vera*	—

Hypertension	Hypertension	*Allium sativum, Annona muricata, Apium graveolens, Petroselinum crispum, Bidens pilosa,Carica papaya, Lagenaria siceraria, Luffa acutangula, Tamarindus indica, Ocimum tenuiflorum, Prunella vulgaris, Moringa oleifera*, *Olea europaea, Crataegus laevigata, Morinda citrifolia, Vangueria madagascariensis, Citrus aurantifolia, Murraya koenigii, *and* Camellia sinensis*	—

Infections and wounds	Ulcers, gangrene, urinary tract infection, and wound healing	*Allium sativum, Brassica oleracea, Cucurbita maxima, Ocimum tenuiflorum, Lysimachia christinae, Cardiospermum halicacabum, *and* Aloe vera*	*Periplaneta americana* *Rattus rattus*

Diabetes	Type 1 diabetes, type 2 diabetes	*Graptophyllum pictum, Allium cepa, Allium sativum, Mangifera indica, Aphloia theiformis, Apium graveolens, Coriandrum sativum, Petroselinum crispum, Catharanthus roseus, Cocos nucifera, Ophiopogon japonicas, Bidens pilosa, Cynara cardunculus, Sigesbeckia orientalis, Brassica oleracea, Cucumis sativus,* *Cucurbita maxima, Lagenaria siceraria, Momordica charantia, Trigonella foenum-graecum, Ocimum tenuiflorum, Linum usitatissimum, Azadirachta indica, Artocarpus heterophyllus, Moringa oleifera, Eucalyptus globules, Psidium guajava, Syzygium cumini,Olea europaea, Phyllanthus emblica, Avena sativa, Rhizophora mucronata, Rubus alceifolius, Morinda citrifolia, Vangueria madagascariensis, Citrus aurantifolia, Citrus maxima, Cardiospermum halicacabum, Camellia sinensis, *and* Aloe vera*	*Salmo salar*

Skin complications	Dry skin	*Avena sativa*	—

**Table 7:** Inventory of animal species used to manage diabetes and related complications.

Class	Scientific name	Local name	Indication	Part used	Method of preparation and administration	RFC	CII_H_	CII_M_	CII_C_	CII_B_
Actinopterygii	*Salmo salar*	Saumon	Type 2 diabetes	Whole body	A dish of the whole body is prepared and it is taken once per week.	0.12	0.04	0.00	0.08	0.00
	*Anguilla japonica*	Anguille	Neuropathic pain	Skin	The skin is peeled and dried in bright sunlight. The dried skin is then placed in a bottle of oil. Massage the painful area daily using this oil.	0.03	0.02	0.00	0.01	0.00

Gastropoda	*Helix aspersa*	Courpa	Cataract	Whole body	The whole body is crushed to obtain white liquid and 2 drops of the liquid are instilled in the eye.	0.02	0.00	0.00	0.02	0.00

Insecta	*Apis mellifera*	Mouche di miel	Cataract	Honey	A small amount is instilled in the eye daily.	0.04	0.01	0.00	0.03	0.00
	*Periplaneta americana*	Cancrela	Gangrene	Whole body	Prepare an infusion with 4 cockroaches and 1 handful of *Petroselinum crispum. *Filter in a cloth and drink 1 cup daily.	0.03	0.00	0.00	0.02	0.01

Mammalia	*Tenrec ecaudatus*	Tang	Renal failure	Whole body	A dish of the body is prepared using *Cinnamomum verum*, *Syzygium aromaticum, Murraya koenigii *L., and 1 cup of white wine. The dish is taken once per week.	0.03	0.00	0.00	0.03	0.00
	*Rattus rattus*	Le rat	Wound	Whole body	The animal is placed in a bottle of coconut oil for 1-2 days and the oil is then applied on the wound.	0.02	0.00	0.00	0.00	0.02

RFC: relative frequency of citation, CII_H_: cultural importance index among the Hindu community, CII_M_: cultural importance index among the Muslim community, CII_C_: cultural importance index among the Christian community, and CII_B_: cultural importance index among the Buddhist community.
