# Supplementary material for: Traditional Therapies Used to Manage Diabetes and Related Complications in Mauritius: A Comparative Ethnoreligious Study
Source: Evid Based Complement Alternat Med. 2016 Apr 21;2016:4523828. doi: 10.1155/2016/4523828 (PMC4856945; doi:10.1155/2016/4523828)
Supplement: Supplementary file 1 — A semi-structured questionnaire was used to seek data from indigenous people. The questionnaire comprised of both open and closed ended questions ranging from demographic data of the respondents to the traditional remedies (herbal and animal-based) used by the indigenous people. [file 4523828.f1.docx]

**Questionnaire**

**Interviewee’s approval agreement.**

I (name is optional) hereby agree to participate in this study with full consent and conscious and declare that all information I’ll provide will be true, accurate and complete.

Date: Signature/thumb of interviewee:

**Please tick in the appropriate box and or fill in words where required**

**SECTION A : DEMOGRAPHIC DATA**

1. Age:

30-39 years old 40-49 years old 50-59 years old

60-69 years old 70-79 years old ≥80 years old

2. Sex: Male Female

3. Level of education:

No formal education Primary

Secondary Tertiary

4. Occupation:

Housewife Retired

Government officer Non-government officer

Traditional healer Ayurvedic medicine practitioner

Traditional Chinese medicine practitioner

5. Monthly household income:

< Rs 5000 Rs 5001-10000 Rs10001-20000

Rs20001-30000 >Rs 30001

6. To which religion to you belong?

Hindu Muslim Christian

Chinese None Other, please specify

7. From which type of diabetes do you suffer *(applicable to diabetic participants)*?

Type 1 diabetes Type 2 diabetes

8. Diabetes entail a number of health complications. Do you suffer from any diabetes related complications confirmed by your treating physician that these complications are related to diabetes *(applicable to diabetic participants)*?

*If no, go to question 9*

Renal failure Eye problems High blood pressure

Cardiovascular disease Sexual dysfunction Urinary tract infections

Skin problems Diabetic neuropathy Ulcers

Diabetic foot Bone and joint disorders None

Other, please specify

**SECTION B : USE OF HERBAL REMEDIES**

9. Do you use herbal remedies to manage diabetes and diabetes related complications?

Yes No

If No, go to section C

10. Which herbal remedies do you use/ have used/ prescribed to manage diabetes and diabetes related complications?

| **Herb name** | **Health problem** | **Part of the plant used** | **The method of preparation** | **Route of administration and dosage** | **Religious virtue of plant** |
| --- | --- | --- | --- | --- | --- |
|  |  |  |  |  |  |
|  |  |  |  |  |  |
|  |  |  |  |  |  |

11. Was there any improvement in your health state after using herbal remedies?

Yes No

12. Do you use polyherbal formulation to manage diabetes and diabetes related complications?

Yes No

*If no, go to question (14)*

13. Which polyherbal formulation do you use/ have used/ prescribed to manage diabetes and related complications?

| **Herbs name** | **Health problem** | **Part of the plants used** | **The method of preparation** | **Route of administration and dosage** |
| --- | --- | --- | --- | --- |
|  |  |  |  |  |
|  |  |  |  |  |
|  |  |  |  |  |

**SECTION C : USE OF ANIMAL PRODUCTS**

14. Do you use animal product(s) for the management of diabetes and diabetes related complications?

Yes No

*If no, end of questionnaire*

15. Please specify the animal product(s) used/ prescribed to manage diabetes and related complications

| **Name of animal** | **Health problem** | **Part of the body used** | **The method of preparation** | **Route of administration and dosage** |
| --- | --- | --- | --- | --- |
|  |  |  |  |  |
|  |  |  |  |  |
|  |  |  |  |  |

16. Was there any improvement in your health state after using animal-based remedies?

Yes No

**Thank you for your participation**
